# Low-Temperature Mechano-Chemical Rubber Reclamation Using Terpinene as a Swelling Agent to Enhance Bond-Breaking Selectivity

**DOI:** 10.3390/polym13244272

**Published:** 2021-12-07

**Authors:** Lei Guo, Donghui Ren, Wenchao Wang, Kuanfa Hao, Xiurui Guo, Fumin Liu, Yuan Xu, Miaomiao Liu, Haichao Liu

**Affiliations:** 1 College of Electromechanical Engineering, Qingdao University of Science & Technology, Qingdao 266061, China; 06weny@163.com (L.G.); 17863950018@163.com (D.R.); wang1436130489@163.com (W.W.); jayhao0730@163.com (K.H.); G847025659@163.com (X.G.); liufumin@qust.edu.cn (F.L.); 2 National Engineering Research Center of Advanced Tire Equipment and Key Materials, Qingdao University of Science & Technology, Qingdao 266061, China; 3 Sino-Thai International Rubber College, Qingdao University of Science & Technology, Qingdao 266061, China; xuyuan@qust.edu.cn; 4 FAW-Volkswagen Automotive Co. Ltd, Changchun 130011, China; miaomiao.liu@faw-vw.com

**Keywords:** rubber reclamation, swelling agent, mechano-chemical devulcanization

## Abstract

Common swelling agents used in the mechano-chemical rubber devulcanization process usually require high temperatures to achieve satisfactory swelling effects, which results in severe production of pollutants and reduces the selectivity of bond scissions. This work presents an environmentally friendly swelling agent, terpinene, which can swell the rubber crosslink structures at low temperatures. Both a rubber swelling experiment and a rubber reclaiming experiment with a mechano-chemical devulcanization method are conducted to explore the swelling effects of terpinene. After soaking in terpinene at 60 °C for 90 min, the length elongation of the rubber sample reaches 1.55, which is much higher than that in naphthenic oil and is comparable to that in toluene. When adding 3 phr of terpinene for every 100 phr of waste rubber during the reclaiming process, the bond scissions exhibit high selectivity. After revulcanization, the reclaimed rubbers have a tensile strength of 17 MPa and a breaking elongation of 400%. Consequently, the application of terpinene as the swelling agent in the LTMD method can greatly improve the properties of reclaimed rubbers, thereby enhancing the dual value for the economy and environment.

## 1. Introduction

With the rapid development of human society, the demand for rubber tires keeps growing. The large increase in demand for rubber tires generates large amounts of tire waste. Nowadays, over three billion tires are produced and one billion tires are abandoned around the world every year [1]. Waste tires do not decompose well due to the crosslinking structure of vulcanized rubber with sulfur bonds [2,3,4], and waste tire piles act as breeding grounds for mosquitoes and other insects that spread disease, especially contributing to the spread of diseases such as malaria and dengue fever. The waste tires still have high value and can be reused/recycled through specific techniques to produce many products, such as playgrounds, sports surfaces, rubber-modified asphalt, and even low-performance tires. Consequently, the disposal of waste tires is an important issue that not only concerns the environment but also economics [5].

Rubber reclamation is an effective method of handling waste rubbers and extracting their value [6,7]. Rubber reclamation is a process of breaking the three-dimensional crosslink structure of waste rubbers and thus obtaining reclaimed rubbers with no or few crosslinks [8]. Common methods for rubber reclamation include thermo-mechanical methods [9,10], mechano-chemical methods [11,12], grinding methods, microwave methods [13,14], and ultrasonic methods [15,16]. Among them, thermo-mechanical methods are most commonly used in the industry and rely on high temperature and mechanical force to achieve devulcanization [17]; however, the high temperature decreases the selectivity of bond scissions, that is, large amounts of C-C bonds are broken during the reclaiming process, which severely weakens the properties of the reclaimed rubbers. Furtherly, serious non-selective bond scissions, together with the oxidation effect, generate unexpected small-molecule substances, including some toxic and harmful gases, resulting in severe environmental pollution.

Mechano-chemical methods are promising alternatives for rubber reclamation as they can combine the effect of mechanical force with chemical reactions and can realize rubber reclamation at lower temperatures than that with thermo-mechanical methods. Rooj et al. [18] and Shi et al. [19] compared the effects of mechano-chemical methods with other methods in terms of the properties of the reclaimed rubbers and found that a mechano-chemical method can significantly enhance the selectivity of bond scissions. Additionally, the low-temperature condition with such a method can reduce the generation of toxic and harmful gases, thereby ensuring cleaner production. Based on the mechano-chemical method, we have developed low-temperature rubber reclaiming equipment (LTRE) in our previous work and obtained reclaimed rubbers with much higher properties than those produced by mechano-chemical methods [20,21].

During rubber reclamation processes, swelling agents are important additives for relaxing the molecular structures of the rubbers and promoting the devulcanization process. Sufficient swelling of the rubber crosslinking structures can enhance the degree of devulcanization and selectivity. Organic solvents may be used, such as alcohol, ketone, and toluene [22], which has a strong swelling effect and can be used as swelling agents [8]; however, the non-economical and non-ecological aspects of organic solvents greatly restrict their applications. Supercritical fluids (water, ethanol, carbon dioxide, and toluene) have also been used for rubber reclamation [23,24]. Asaro et al. [25] conducted a rubber reclamation experiment in a supercritical carbon dioxide (scCO_2_) atmosphere. The maximum degree of devulcanization reached 90% and they attributed this result to the excellent swelling effect of the scCO_2_. A hydrothermal process for subcritical fluids shares similar properties with supercritical fluids regarding solvating and a swelling agent [26]. The subcritical fluid can promote the devulcanization reaction, increase the bond-breaking selectivity, prevent oxidative degradation in materials, and significantly improve the mechanical properties of the re-vulcanizates. Among different subcritical fluids (e.g., water, ethanol, and propanol), the influence of subcritical ethanol is significantly obvious [27]. Thermoplastic polymers can also swell the crosslinking structures of rubbers [28]. Barbosa et al. [28] added thermoplastic ethylene vinyl-acetate during rubber reclamation using an intermeshing co-rotating twin-screw extruder. The addition of the ethylene vinyl-acetate achieved effective devulcanization, evidenced in the increase of soluble fractions, reduction in crosslinking density, and thermal stability gain.

Oil liquids, such as naphthenic oil and turpentine, are the most widely used swelling agents for rubber reclamation because they require simpler process conditions than the supercritical fluids and subcritical fluids and feature environmental and economic advantages over organic solvents. Li et al. [29] thermo-mechanically devulcanized ground tire rubber (GTR) via extrusion using waste engine oil (WEO) as the swelling agent. They found that the WEO had a positive effect on the swelling of the GTR and regarding the fractures of partial crosslinking bonds. Sabzekar et al. [30] mechano-chemically devulcanized waste EPDM rubber under temperatures ranging from 220 °C to 290 °C using disulfide oil (DSO), which is an oily waste produced in gas refineries, as the swelling agent. The DSO swelling agent could decrease the crosslinking density by up to 73% at specific reaction conditions. Though the oil liquids show good swelling effects, most of them require high temperatures to accelerate their dipping and swelling processes, which cannot fully meet the requirements of a low-temperature mechano-chemical devulcanization (LTMD) process.

In this work, we present a new swelling agent, terpinene, which has an excellent swelling effect on vulcanized rubber at low temperatures. This discovery came from reflection on the fact that lemon peel juice dripping on a balloon would cause a balloon to burst. We tested different peel juice samples to explore their effect on balloons and analyzed the main components in the peel juice which could cause a balloon to burst, finally finding that terpinene (C_10_H_16_) is responsible for causing the balloon to burst. Terpinene has three common isomers, namely, α-terpinene, β-terpinene, and γ-terpinene, of which γ-terpinene exists in lemon peel juice. Notably, γ-terpinene is a colorless liquid with citrus and lemon aromas. It is soluble in ethanol and most non-volatile oils but is insoluble in water. γ-terpinene naturally exists in coriander seed oil, lemon oil, cumin oil, and parsley oil, and it can also be separated from the essential oil of *Lantana camara*.

γ-terpinene is a monoterpene that is a monocyclic compound which has an alternating structure of single and double bonds, similar to natural rubber, so it may have good compatibility with natural rubber. Additionally, its small molecular weight may accelerate its infiltration into natural rubber. To explore the swelling effect of γ-terpinene, we conducted swelling and rubber reclamation experiments using γ-terpinene as the swelling agent. The characteristics and properties of the reclaimed rubbers were analyzed and the effect of γ-terpinene on production efficiency was investigated. All the descriptions of “terpinene” in the subsequent context refer to γ-terpinene.

## 2. Materials and Methods

### 2.1. Materials

**Figure 1:**
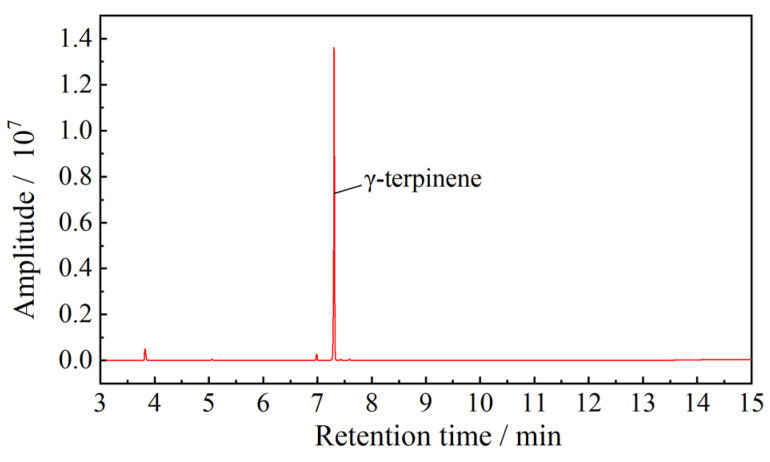
Testing results of the terpinene material using a gas chromatograph.

This work used waste tire tread strips (Shandong New Dongyue Renewable Resources Technology Co., Ltd, Qingdao, Shandong, China), self-prepared rubber sheets vulcanized using natural rubber, terpinene (CAS: 99-85-4, Jiangshan polymer material Co., Ltd, Dongying, Shandong, China), an activator (2,4-di-tert-butylphenol, CAS: 96-97-4, Fuxin Chemical Technology Service Co., Ltd, Shanghai, China), Tetraethylenepentamine (TEPA, CAS: 112-57-2, Fuchen Chemical Reagent Co., Ltd, Tianjin, China), and other commercially available assistants, such as zinc oxide (ZnO), stearic acid, sulfur, and an accelerator (N-tert-Butyl-2-benzothiazolesulfenamide, TBBS, CAS: 95-31-8). The components of the purchased terpinene material were tested using a gas chromatograph. The testing result is shown in Figure 1 and the purity of γ-terpinene was identified to be 95.4%.

### 2.2. Experimental Procedure

#### 2.2.1. Swelling

The self-prepared vulcanized rubber sheets with the shape of 20 × 20 × 2 mm^3^ were swelled in terpinene, toluene, and naphthenic oil at the temperatures of 30 °C, 60 °C, or 90 °C, respectively, to explore their swelling effects. Length elongations of the rubber samples were recorded after soaking in the swelling agent for 10 min, 30 min, 60 min, and 90 min, respectively.

#### 2.2.2. Rubber Reclamation

Waste tire tread strips were first added into the internal mixer of the LTRE [21] and mixed at the roller speed of 50 r min^−1^ and barrel temperature of 40 °C for 25 s. Then, terpinene and TEPA were added into the internal mixer and the barrel temperature was increased to 80 °C for 20 min. After that, the rubber compounds were transported into the twin-screw extruder of the LTRE, which was operated at a screw speed of 50 r min^−1^ and barrel temperature of 50 °C. The extruded rubber compounds were then thin-passed 15 times in the finishing mill.

The addition amount of terpinene was considered in the rubber reclamation process, which included 0, 0.5, 1, 2, 3, and 4 phr in every 100 phr of reclaimed rubber. TEPA was fixed at 3 phr for every 100 phr of reclaimed rubber.

#### 2.2.3. Mixing and Vulcanization

The re-vulcanization of the reclaimed rubbers requires a mixing process to mix the reclaimed rubbers with additives. The re-vulcanization formula consisted of the reclaimed rubber, sulfur, stearic acid, ZnO, and an accelerator at a mass ratio of 300:3.5:1:7.5:2.4. The mixing process was operated in an open mill with a roller spacing of 1.8 mm. The re-vulcanization process was conducted under the optimum condition at 150 °C.

### 2.3. Measurements and Characterizations

- Sol Fraction

A rubber sample with a specific mass was first extracted with acetone in a Soxhlet extraction for 12 h and subsequently dried to a constant weight ($m_{1}$) in a vacuum drying oven at 80 °C. Then, it was extracted with toluene for 16 h and subsequently dried to a constant weight ($m_{2}$) in a vacuum drying oven at 80 °C. The sol fraction was obtained from the weight of the rubber sample before and after extraction as follows:

(1) $$S_{f}=(m_{1}-m_{2})/m_{1}$$

- Crosslinking Density and Devulcanization Ratio

The equilibrium swelling method with the Flory–Rehner equation [19] was used to calculate the crosslinking density values of the rubber samples. A small piece of a rubber sample was first extracted with acetone in a Soxhlet extraction for 12 h and subsequently dried to a constant weight ($m_{i}$) in a vacuum drying oven at 80 °C. Then, the sample was swollen in toluene at ambient temperature for 72 h, weighed ($m_{t}$) after removal from the toluene, and subsequently dried to a constant weight ($m_{d}$) in a vacuum drying oven at 80 °C. Then, the crosslinking density of the rubber sample could be calculated by the following formula:

(2) $$v_{e}=\frac{-\left[\ln\left(1-V_{r}\right)+V_{r}+\chi V_{r}{}^{2}\right]}{V_{l}\left(V_{r}{}^{1/3}-V_{r}/2\right)}$$

where $V_{r}$, χ, and $V_{l}$ are the rubber volume fraction in the swollen sample, the rubber-solvent interaction parameter, which was set to 0.43, and the molar volume of toluene, respectively. Among them, the rubber volume fraction $V_{r}$ can be calculated as follows:

(3) $$V_{r}=\frac{m_{d}/\rho_{d}}{m_{d}/\rho_{d}+m_{s}/\rho_{s}}$$

where $m_{d}$ and $\rho_{d}$ are, respectively, the mass and density of the rubber sample after extraction and drying and $m_{s}$ and $\rho_{s}$ are, respectively, the mass of toluene absorbed by the sample and its density at room temperature. The mass of the absorbed toluene can be calculated as follows:

(4) $$m_{s}=m_{t}-m_{d}$$

The devulcanization ratio that indicates the devulcanization degree can be calculated as follows:

(5) $$Rd=\left(\nu_{e1}-\nu_{e2}\right)/\nu_{e1}$$

where Rd denotes the devulcanization ratio and $\nu_{e1}$ and $\nu_{e2}$ denote the crosslinking density of the vulcanized rubber sample and the reclaimed rubber sample, respectively.

#### Gel Permeation Chromatography (GPC)

GPC analysis was used to test the number-average molecular weight via a gel permeation chromatograph (GPC515-2410 System, produced by Waters, Milford, MA, USA). The test used tetrahydrofuran as the mobile phase at the mobile velocity of 1 mL min^−1^ and a temperature of 30 °C. Polystyrene was used as the standard.

- Mooney Viscosity

A Mooney viscometer (UM-2050, Youken Technology Co., Ltd., Taibei, Taiwan, China) was used to test the Mooney viscosity based on the ASTM D 1646 standard (ML 100 °C (1 + 4) min). The rubber samples were preheated for 1 min and then the analysis was conducted at a rotor speed of 2 r min^−1^ and temperature of 100 °C for 4 min. The result was expressed in Mooney units (MU).

- Curing Characteristics

A rotorless rheometer (MDR2000, Alpha Corporation, Akron, OH, USA) was used to characterize the curing characteristics of the reclaimed rubbers, including the maximum torque (MH), minimum torque (ML), scorch time (T10), and optimum cure time (T90). Rubber samples with a weight of 5 g were used and the test was conducted at a temperature of 150 °C.

- Physical Properties

A tension tester (UT-2060, Taiwan Youken Technology Co., Ltd, Taibei, Taiwan, China) was used to test the tensile strength (Rm), breaking elongation (ε), 100% modulus (100% E), and 300% modulus (300% E) values according to the ASTM-D412 standard. The hardness (Shore A) was examined on a Shore A Hardness Tester according to the ASTM-D2240 standard.

- Dynamic Mechanical Properties

A dynamic mechanical analyzer (EPLEXOR 150N, produced by NETZSCH Corporation, Selb, Bavaria, Germany) was used to test the dynamic mechanical properties of the rubber samples using temperature sweeping in tensile mode. The frequency was 10 Hz, the static strain was 5%, the static profit was 70 N, the dynamic strain was 0.25%, the dynamic stress was 60 N, the temperature range was from −65 to 65 °C, and the heating rate was 2 °C min^−1^.

## 3. Results and Discussion

### 3.1. Swelling Effect of Terpinene on Vulcanized Rubbers

**Figure 2:**
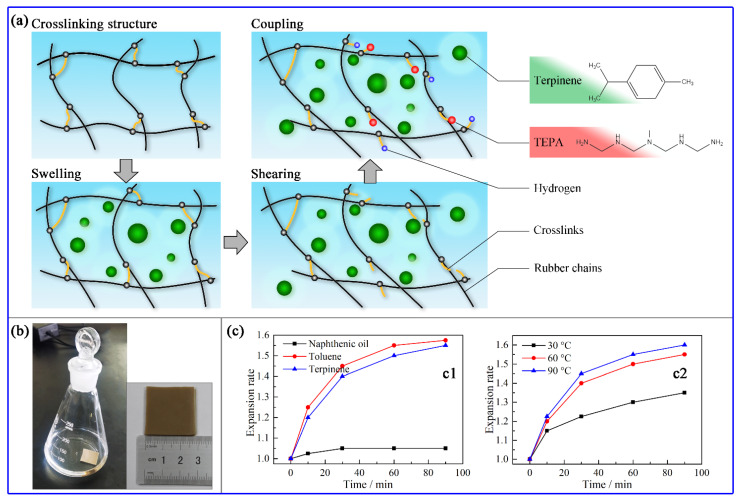
(**a**) Devulcanization mechanism of the LTMD method with the effect of swelling agents, devulcanizing agents, and shearing force. (**b**) Swelling processes of the rubber samples in swelling agents at 60 °C. (**c**) Length elongation of rubber samples with different swelling agents at 60 °C (**c1**) and under different temperatures (**c2**) as functions of swelling time.

The devulcanization process of waste rubbers via the LTMD method includes three steps (Figure 2a). Firstly, the uniformly distributed and dispersed swelling agents swell the rubber crosslinking structures, reducing the degree of entanglement and weakening the intermolecular forces. Secondly, the mechanical shearing forces stretch the rubber molecules and break some of the chemical bonds of the rubber molecules. Since the bond energies of the S-S and S-C bonds are lower than those of the C-C bonds, the crosslinking bonds are easier to break than those of the main chains. More importantly, the swelling effect of the swelling agents greatly enhances this kind of bond-breaking selectivity. Lastly, the positively charged –S+ and negatively charged –S− generated from the S-S scissions are coupled with TEPA, thereby preventing their reattachment [31]. The N atoms of the secondary amine in TEPA possess lone pair electrons and thus can combine with –S+. Meanwhile, the proton H of the secondary amine in TEPA is released and can combine with –S−. In this way, the waste rubbers are devulcanized with high bond-breaking selectivity and the reclaimed rubbers have feature a low Mooney rebound effect.

The swelling experiment and length elongations of the vulcanized rubbers are shown in Figure 2b,c, respectively. The rubber sample immersed in naphthenic oil expanded before 30 min and then became stable, where the length elongation rate was around 1.05. The rubber sample immersed in terpinene continually expanded as time went on. Its length elongation rate was around 1.55 after 90 min, close to the value of 1.575 in toluene. The excellent swelling effect of terpinene is related to its molecular structure, which is mainly composed of C-C, C=C, and C-H bonds. These compositions are similar to those in rubbers, thus, terpinene shows close polarity and good compatibility to vulcanized rubbers. Consequently, terpinene can easily infiltrate the network structure of vulcanized rubber and lead to its expansion. Compared with naphthenic oil, terpinene has a much lower molecular weight and thus a better infiltration effect. 

The swelling effect of terpinene is significantly affected by temperature. As shown in Figure 2c, with the temperature increasing from 30 °C to 60 °C, the expansion rates of the rubber samples increased largely, and with a temperature increasing from 60 °C to 90 °C, the expansion rates of the rubber samples increased slightly. As such, the optimum working temperature for terpinene ranges from 60 °C to 90 °C, which is suitable for the LTMD process using the LTRE.

### 3.2. Effect of Terpinene on Bond Scissions during the Rubber Reclamation Process

It has been confirmed in the above context that terpinene has a good swelling effect on vulcanized rubbers. To verify its effect on the rubber reclamation process, we carried out rubber reclamation experiments on the LTRE with the LTMD method, in which terpinene was used as the swelling agent. Characteristics of the reclaimed rubbers, including the number-average molecular weight, sol fraction, Mooney viscosity, and devulcanization ratio, were analyzed in order to explore the bond-breaking degree and selectivity.

#### 3.2.1. Characteristics of the Reclaimed Rubbers

Characteristics of the reclaimed rubbers devulcanized with different amounts of terpinene, including the number-average molecular weight, sol fraction, Mooney viscosity, and devulcanization ratio, are shown in Table 1. The data shown in the table are the average values of three experiments. With the increase of terpinene, the sol fraction and devulcanization ratio increases and the Mooney viscosity decreases. These results demonstrate that the devulcanizing degree is enhanced and crosslink bonds are increasingly broken. This enhancement is attributed to the increase of terpinene, which improves the swelling effect, weakens the intermolecular forces, and thus heightens the effect of the mechanical shearing forces on bond scissions.

The enhancement of the devulcanizing degree should have caused the number-average molecular weight to decrease. Conversely, the number-average molecular weight of the reclaimed rubbers increased with the increase of terpinene below 3 phr. This result indicates that, as the terpinene amount increases, the amount of main chain scissions does not rise synchronously with that of the crosslink scissions. The most possible result is that the increase of terpinene decreases the main chain scissions and increases the crosslink scissions. The retention of the main chains increases the number-average molecular weight of the reclaimed rubbers and the crosslink scissions increase the Sol fraction and decrease the Mooney viscosity, where, as a result, the devulcanization ratio is improved.

When terpinene exceeded 3 phr, the number-average molecular weight decreased sharply. This is probably because terpinene with 4 phr causes an excessive swelling effect, which leads to a great increase of C-C scissions under the action of mechanical forces. As a result, the main chains of the reclaimed rubbers are shortened and the number-average molecular weight decreases.

#### 3.2.2. Horikx

**Figure 3:**
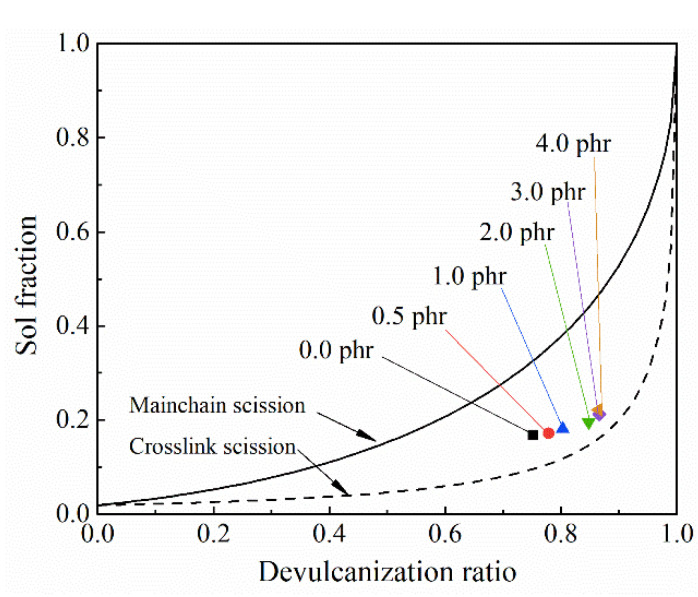
Horikx curves indicating the bond-breaking types according to the relations between the devulcanization ratio and sol fraction (the dashed line represents only crosslink scissions, and the solid line represents only C-C scissions.).

The amounts of main chain scissions and crosslink scissions reflect the bond-breaking selectivity of the rubber reclamation process. Horikx theory [32] was used by establishing the relationships between the devulcanization ratios and sol fractions to exhibit the bond-breaking selectivity intuitively, as shown in Figure 3. The dashed line in the figure corresponds to more selective crosslink scissions, and the solid line corresponds to mainchain scissions and polymer degradation. When no terpinene was added during the LTMD process, the point was located in the middle of the main chain scission line and crosslink scission line, indicating that the proportions of main chain scissions and crosslink scissions were close. With the increase of terpinene below 3 phr, the point gradually moved close to the crosslink scission line, indicating that the bond-breaking selectivity was improved; however, when terpinene exceeded 3 phr, the point moved a little toward the main chain scission line, indicating that the bond selectivity decreased slightly. This result is consistent with the analysis result of the characteristics of the reclaimed rubbers in the above context (Table 1). This result confirms that terpinene is efficient for the devulcanization in a rubber reclamation process and that the addition amount of terpinene should be 3 phr in every 100 phr of waste rubber to maximum the devulcanization effect.

The improvement of the bond-breaking selectivity with the increase of terpinene is mainly attributed to the enhanced swelling effect. With the increase of terpinene, the swelling of the rubber molecular structures is improved and thus the mechanical shearing force can better act on and break the S-S bonds or S-C bonds, thereby retaining the integrity of the main chains. Additionally, terpinene has a lubricating effect, which can make the distribution of the shearing force uniform and decrease the local excessive shearing force, thereby reducing the probability of the C-C scissions. Consequently, terpinene of appropriate amount can promote the selectivity of bond scissions, lengthening the molecular chains of the reclaimed rubbers and simultaneously deepening devulcanization degree, which cause the molecular structures of the reclaimed rubbers closer to that of the original rubbers before vulcanization.

### 3.3. Properties of Reclaimed Rubbers

The high devulcanization degree and selectivity attributed to the effect of terpinene are probably conducive to the properties of the reclaimed rubbers. In this section, the curing characteristics, physical properties, and dynamic mechanical properties of the reclaimed rubbers are carefully investigated.

#### 3.3.1. Curing Characteristics

The curing characteristics of the reclaimed rubbers devulcanized with different amounts of terpinene are shown in Table 2. The data in the table show the average values of three experiments. Scorch time (T10), optimum cure time (T90), minimum torque (ML), maximum torque (MH), the difference between MH and ML (MD) of the reclaimed rubbers all decreased with the increase of terpinenes. The decrease of T10 and T90 may be attributed to the uniform dispersion of terpinene, which increases the flexibility and flowability of the rubber materials and thus causes the curing additives to distribute more uniformly, thereby accelerating the curing process.

The decrease of the ML is due to the fractures of the crosslink bonds, which convert the three-dimensional structure of waste rubbers into chain structure, thereby enhancing the flow and plasticity of the reclaimed rubbers. The MD is calculated by subtracting the ML from the MH, which can reflect the degree of the crosslinking density. The decrease of the crosslinking density is also due to the increased fractures of the crosslink bonds. The sulfur radicals generated by crosslink scissions couple with TEPA and cannot largely re-crosslink. As a result, after the rubber re-vulcanization with the same curing systems, the crosslinking densities of the revulcanized rubbers are lower in cases with larger amounts of terpinene. This result does not mean that the addition of terpinene has a bad effect on the reclaimed rubbers because the addition of terpinene enhances the bond-breaking selectivity and increases the length of the reclaimed rubbers, which is conducive to the improvement of processing and physical properties.

#### 3.3.2. Physical Properties

Table 3 shows the mechanical properties of the re-vulcanized rubbers, for which the analysis was repeated three times. With the increase of terpinene, the tensile strength and breaking elongation of the revulcanized rubbers increases first and then decreases. The increase of the tensile strength and breaking elongation is attributed to the improvement of the bond-breaking selectivity, which improves the number-average molecular weight and enhances the integrity of the main chains; however, with a further increase of terpinene exceeding 3 phr, intermolecular interactions are weakened excessively and the scissions of the main chains increase instead of decreasing under the action of the shearing force, that is, both the mainchain scissions and the crosslink scissions are considerable and the number-average molecular weight decreases seriously (Table 1). Consequently, both the tensile strength and breaking elongation of the revulcanized rubbers are reduced.

Modulus and hardness are two indexes related to the stiffness of the vulcanized rubbers. They have good correlation and their changing rules are consistent. Generally, the modulus and hardness change with the variation of the crosslinking density. As shown in Table 3, the values of the 100% modulus, 300% modulus, and hardness (Shore A) are reduced with the increase of terpinene, almost consistent with the changing tendency of the MD index for the above curing characteristics (Table 2).

When adding 3 phr of terpinene for every 100 phr of waste rubber, the reclaimed rubber possesses the best tensile strength of 17.03 MPa and a breaking elongation of 400.7%. This value of tensile strength is much higher than that found in other work, such as 12.90 MPa [33] and 11.00 MPa [17], which are relatively high tensile strength for of the obtained reclaimed rubbers we found in the literature. The breaking elongation in this work is also higher than that in most literature [34,35,36]. The excellent values of the tensile strength and breaking elongation in this work indicate an outstanding application prospect of the LTMD method with terpinene as the swelling agent.

#### 3.3.3. Dynamic Mechanical Properties

**Figure 4:**
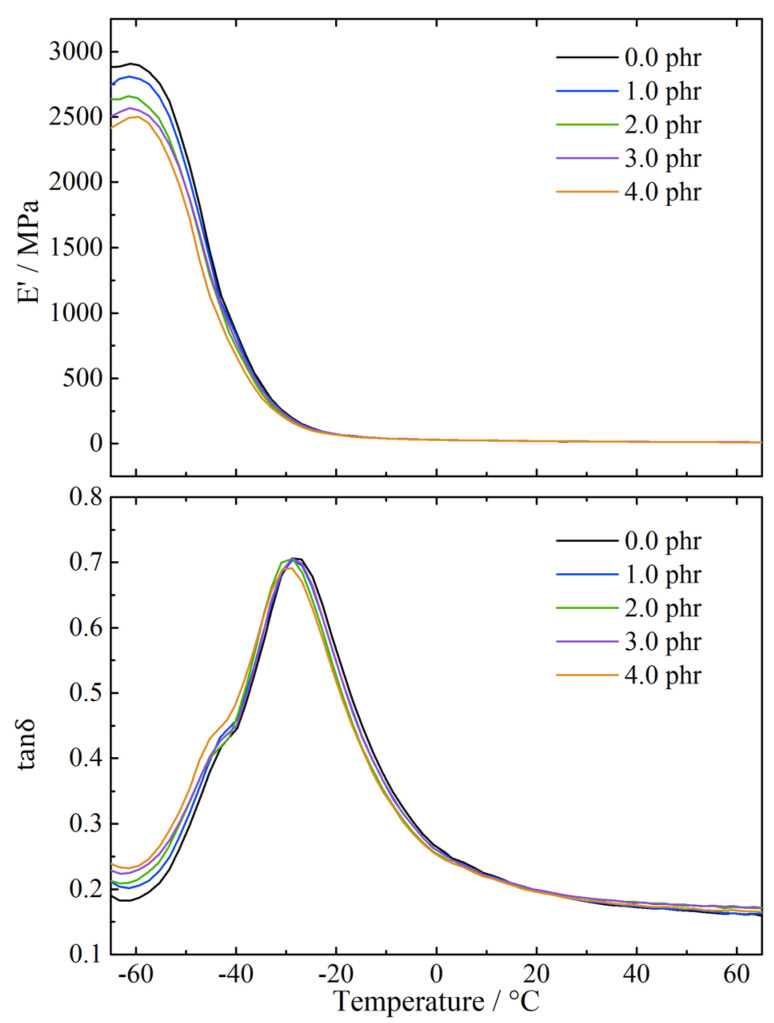
Temperature dependence on E′ and tanδ for revulcanized rubbers devulcanized with different amounts of terpinene.

Figure 4 shows the dynamic mechanical properties of the revulcanized rubbers, including the storage modulus and tanδ. It can be seen that the storage modulus of the revulcanized rubber decreases with the increase of terpinene at low temperatures. This is because the rubber molecular chains are frozen at low temperatures, and the amount of terpinene has a significant effect on the storage modulus. With the increase of terpinene, the crosslinking density of the reclaimed rubbers decreases, and the flexibility of the molecular chains is improved, thereby causing the revulcanized rubbers to be softer and the storage modulus to be lower.

For tanδ, with the increase of terpinene, the curve in the ranges of the glass transition temperature shifts to the left with terpinene below 3 phr and returns to the right with terpinene exceeding 3 phr. The shift of the curve represents the variation of the glass transition temperature, which is related to the length of the molecular chains. With terpinene below 3 phr, the increase of terpinene enhances the bond-breaking selectivity and increases the length of the molecular chains of the reclaimed rubbers. Longer molecular chains are harder to crystallize than shorter molecular chains and thus the glass transition temperature decreases, and the curve shifts to the left. When terpinene reaches 4 phr, the excessive swelling effect causes large amounts of mainchain scissions and thus the molecular chains are shorter than that with terpinene of 3 phr. As a result, the glass transition temperature increases, and the curve returns toward the right. The glass transition temperature reflects the low-temperature resistance of the rubbers. Therefore, the revulcanized rubbers reclaimed with terpinene of 3 phr possess the best low-temperature resistance.

### 3.4. Production Efficiency

**Figure 5:**
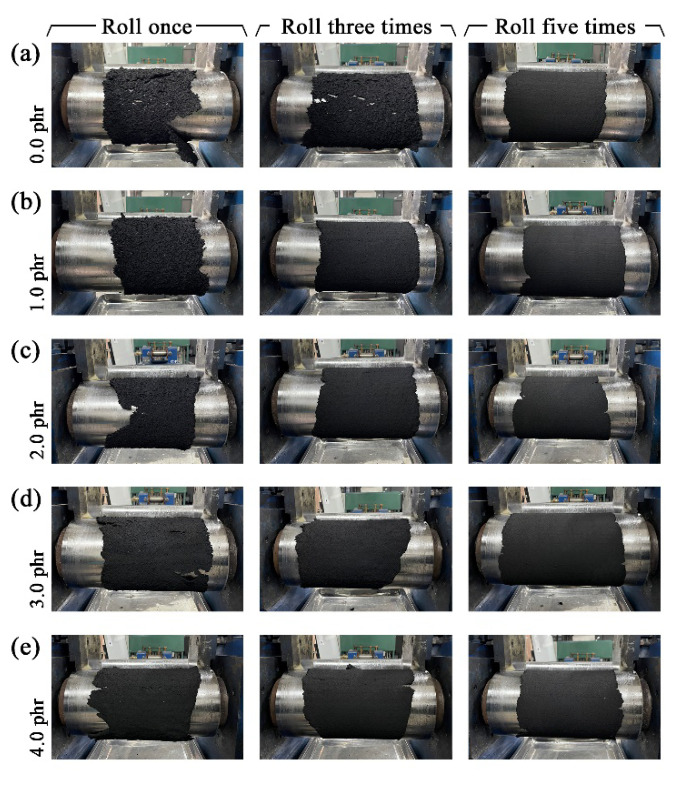
Roller coating status of the reclaimed rubbers devulcanized with different amounts of terpinene, including 0.0 phr (**a**), 1.0 phr (**b**), 2.0 phr (**c**), 3.0 phr (**d**) and 4.0 phr (**e**), during the refining process with different rolling times.

The application of terpinene as the swelling agent showed a positive effect on the properties of the reclaimed rubbers here, which is conducive to enhancing production efficiency. Figure 5 shows the roller coating status of the reclaimed rubbers during the refining process with different rolling times. It can be seen that under the same rolling times, the viscosity of the reclaimed rubber became better with an increase in terpinene. In Figure 5a, it takes five repetitions for the reclaimed rubber to wrap around the roller with no terpinene during the reclamation process, while, in Figure 5d,e, it only takes one repetition for the reclaimed rubber to wrap the roller. This result indicates that, under the same requirement of the reclaimed rubbers, fewer rolling repetitions are required when adding more terpinene. Consequently, the application of terpinene as a swelling agent can also be used to enhance production efficiency and save on costs.

## 4. Conclusions

In this work, the swelling effect of terpinene was confirmed via swelling experiments and its performance on rubber devulcanization in the LTMD method was investigated via reclamation experiments. Terpinene shows an excellent swelling effect below 100 °C, which is much better than traditional naphthenic oil and is comparable to toluene. When used to devulcanize waste rubbers with the LTMD method, terpinene can infiltrate into and relax the rubber crosslink structures, weaken the intermolecular forces, and significantly enhance the bond-breaking selectivity under the action of mechanical shearing forces, thereby improving the properties of reclaimed rubbers. An excessive addition of terpinene may cause excessive swelling, leading to an increase in mainchain scissions, which conversely reduces the properties of the reclaimed rubbers. As a result, the tensile strength and breaking elongation of the revulcanized rubbers increases first and then decreases with an increase in terpinene. The optimum mass ratio of terpinene was found to be 3 phr for every 100 phr of waste rubber, with which the revulcanized rubber possesses a tensile strength of 17 MPa, a breaking elongation of 400%, and resistance to low temperatures. Furthermore, the addition of terpinene can also shorten the milling time due to the high devulcanization ratio, thus improving production efficiency and saving on costs. Terpinene has bright prospects in rubber reclaiming applications using LTMD methods because of its environmental friendliness and excellent swelling effect at low temperatures.

The terpinene material used in this work was γ-terpinene. In future work, we will further investigate the swelling effect of different isomers of terpinene and try to explore the swelling mechanism at the molecular scale.

## Figures and Tables

**Table 1:** The number-average molecular weight (Mn), sol fraction, Mooney viscosity, and devulcanization ratio as functions of the terpinene amount.

Terpinene Amount/phr	Mn	Sol Fraction	Mooney Viscosity	Devulcanization Ratio
0.0	18,987	16.7%	105.7	75.2%
0.5	20,540	17.1%	104.7	77.9%
1.0	20,750	18.0%	103.5	80.3%
2.0	20,964	19.6%	97.1	84.8%
3.0	21,484	21.2%	90.3	86.6%
4.0	19,570	22.1%	87.4	86.5%

**Table 2:** Curing characteristics of the reclaimed rubbers devulcanized with different amounts of terpinene. (T10: scorch time; T90: optimum cure time; ML: minimum torque; MH: maximum torque; MD: the difference between MH and ML. The terpinene amount represents the adding amount of terpinene for every 100 phr of waste rubber.

Terpinene Amount/phr	T10/Min	T90/Min	ML/N m	MH/N m	MD/N m
0.0	0.63	8.91	2.80	13.78	10.98
0.5	0.63	8.67	2.71	13.59	10.88
1.0	0.56	6.97	2.67	13.30	10.63
2.0	0.53	6.44	2.66	12.66	10.01
3.0	0.51	5.89	2.64	12.13	9.49
4.0	0.50	5.50	2.60	12.01	9.41

**Table 3:** Mechanical properties, including the tensile strength, breaking elongation, 100% modulus (100% E), 300% modulus (300% E), and hardness (Shore A), of the revulcanized rubbers devulcanized with different adding amounts of the terpinene swelling agent.

Terpinene Amount/phr	Tensile Strength/MPa	Breaking Elongation/%	100% Modulus/MPa	300% Modulus/MPa	Hardness (Shore A)
0.0	15.52 ± 0.54	325.5 ± 6.6	4.10 ± 0.03	14.23 ± 0.24	75.5 ± 0.5
0.5	16.36 ± 0.53	351.9 ± 12.0	3.83 ± 0.10	13.95 ± 0.25	75.0 ± 0.5
1.0	16.73 ± 0.77	357.7 ± 17.9	3.81 ± 0.07	13.79 ± 0.25	74.5 ± 0.5
2.0	16.81 ± 0.35	365.7 ± 12.4	3.47 ± 0.08	12.51 ± 0.17	73.5 ± 1.0
3.0	17.03 ± 0.64	400.7 ± 23.4	3.39 ± 0.12	11.95 ± 0.70	73.0 ± 1.0
4.0	15.64 ± 0.47	387.5 ± 33.9	3.17 ± 0.13	11.54 ± 0.56	72.5 ± 0.5

## References

[1] Bowles A.J., Fowler G.D., O’Sullivan C., Parker K. (2020). Sustainable rubber recycling from waste tyres by waterjet: A novel mechanistic and practical analysis. SM&T.

[2] Liu H.L., Wang X.P., Jia D.M. (2020). Recycling of waste rubber powder by mechano-chemical modification. J. Clean Prod..

[3] El-Nemr K.F., Raslan H.A., Ali M.A.M., Hasan M.M. (2020). Innovative gamma rays irradiated styrene butadiene rubber/reclaimed waste tire rubber blends: A comparative study using mechano-chemical and microwave devulcanizing methods. J. Polym. Eng..

[4] Hassan M.M., Badway N.A., Elnaggar M.Y., Hegazy E.S.A. (2013). Thermo-mechanical properties of devulcanized rubber/high crystalline polypropylene blends modified by ionizing radiation. J. Ind. Eng. Chem..

[5] Sathiskumar C., Karthikeyan S. (2019). Recycling of waste tires and its energy storage application of by-products—A review. SM&T.

[6] Wang Z.F., Zeng D.P. (2021). Preparation of devulcanized ground tire rubber with supercritical carbon dioxide jet pulverization. MatL.

[7] Hassan M.M., Aly R.O., Aal S.E.A., El-Masry A.M., Fathy E.S. (2013). Styrene butadiene-based blends containing waste rubber powder: Physico-mechanical effects of mechanochemical devulcanization and gamma irradiation. J. Ind. Eng. Chem..

[8] Bockstal L., Berchem T., Schmetz Q., Richel A. (2019). Devulcanisation and reclaiming of tires and rubber by physical and chemical processes: A review. J. Clean Prod..

[9] Formela K., Wasowicz D., Formela M., Hejna A., Haponiuk J. (2015). Curing characteristics, mechanical and thermal properties of reclaimed ground tire rubber cured with various vulcanizing systems. Iran. Polym. J..

[10] Gagol M., Boczkaj G., Haponiuk J., Formela K. (2015). Investigation of volatile low molecular weight compounds formed during continuous reclaiming of ground tire rubber. Polym. Degrad. Stab..

[11] Sripornsawat B., Saiwari S., Pichaiyut S., Nakason C. (2016). Influence of ground tire rubber devulcanization conditions on properties of its thermoplastic vulcanizate blends with copolyester. Eur. Polym. J..

[12] Hassan M.M., Aly R.O., Aal S.E.A., El-Masry A.M., Fathy E.S. (2013). Mechanochemical devulcanization and gamma irradiation of devulcanized waste rubber/high density polyethylene thermoplastic elastomer. J. Ind. Eng. Chem..

[13] Formela K., Hejna A., Zedler L., Colom X., Canavate J. (2019). Microwave treatment in waste rubber recycling-recent advances and limitations. Express Polym. Lett..

[14] Simon D.A., Pirityi D., Tamas-Benyei P., Barany T. (2020). Microwave devulcanization of ground tire rubber and applicability in SBR compounds. J. Appl. Polym. Sci..

[15] Elnaggar M.Y., Fathy E., Amdeha E., Hassan M.M. (2019). Impact of gamma irradiation on virgin styrene butadiene rubber blended with ultrasonically devulcanized waste rubber. Polym. Eng. Sci..

[16] Sun X.M., Isayev A.I. (2008). Continuous ultrasonic devulcanization comparison of carbon black filled synthetic isoprene and natural rubbers. Rubber Chem. Technol..

[17] Seghar S., Asaro L., Rolland-Monnet M., Hocine N.A. (2019). Thermo-mechanical devulcanization and recycling of rubber industry waste. Resour. Conserv. Recycl..

[18] Rooj S., Basak G.C., Maji P.K., Bhowmick A.K. (2011). New Route for Devulcanization of Natural Rubber and the Properties of Devulcanized Rubber. J. Polym. Environ..

[19] Shi J., Jiang K., Ren D., Zou H., Wang Y., Lv X., Zhang L. (2013). Structure and performance of reclaimed rubber obtained by different methods. J. Appl. Polym. Sci..

[20] Guo L., Lv D., Ren D., Qu L., Wang W., Hao K., Guo X., Chen T., Sun J., Wang C. (2021). Effectiveness of original additives in waste rubbers for revulcanization after reclamation with a low-temperature mechanochemical devulcanization method. J. Clean Prod..

[21] Guo L., Wang C., Lv D., Ren D., Zhai T., Sun C., Liu H. (2021). Rubber reclamation with high bond-breaking selectivity using a low-temperature mechano-chemical devulcanization method. J. Clean Prod..

[22] De Sousa F.D.B., Scuracchio C.H., Hu G.H., Hoppe S. (2017). Devulcanization of waste tire rubber by microwaves. Polym. Degrad. Stab..

[23] Asaro L., Gratton M., Seghar S., Hocine N.A. (2018). Recycling of rubber wastes by devulcanization. Resour. Conserv. Recycl..

[24] Meysami M., Tzoganakis C., Mutyala P., Zhu S.H., Bulsari M. (2017). Devulcanization of Scrap Tire Rubber with Supercritical CO2: A Study of the Effects of Process Parameters on the Properties of Devulcanized Rubber. Int. Polym. Proc..

[25] Asaro L., Gratton M., Poirot N., Seghar S., Hocine N.A. (2020). Devulcanization of natural rubber industry waste in supercritical carbon dioxide combined with diphenyl disulfide. Waste Manag..

[26] Wang X.J., Shi C.P., Zhang L., Zhang Y.C. (2013). Effects of shear stress and subcritical water on devulcanization of styrene-butadiene rubber based ground tire rubber in a twin-screw extruder. J. Appl. Polym. Sci..

[27] Zhang T., Cao J.X., Wang X.J., Zhang L., Zhang Y.C. (2018). Influences of process variables and subcritical fluids on epdm-assisted devulcanization of sbr-based ground tire rubber. Rubber Chem. Technol..

[28] Barbosa R., Nunes A.T., Ambrosio J.D. (2017). Devulcanization of Natural Rubber in Composites with Distinct Crosslink Densities by Twin-Screw Extruder. Mater. Res-Ibero-Am. J..

[29] Li Y., Shen A.Q., Lyu Z.H., Wang S.F., Formela K., Zhang G.T. (2019). Ground tire rubber thermo-mechanically devulcanized in the presence of waste engine oil as asphalt modifier. Constr. Build. Mater..

[30] Sabzekar M., Zohuri G., Chenar M.P., Mortazavi S.M., Kariminejad M., Asadi S. (2016). A new approach for reclaiming of waste automotive EPDM rubber using waste oil. Polym. Degrad. Stab..

[31] Jiang C., Zhang Y.S., Ma L., Zhou L., He H. (2018). Tailoring the properties of ground tire rubber/high-density polyethylene blends by combining surface devulcanization and in-situ grafting technology. MCP.

[32] Horikx M.M. (1956). Chain scissions in a polymer network. J. Polym. Sci..

[33] Tao G.L., He Q.H., Xia Y.P., Jia G.C., Yang H.C., Ma W.Z. (2013). The effect of devulcanization level on mechanical properties of reclaimed rubber by thermal-mechanical shearing devulcanization. J. Appl. Polym. Sci..

[34] Ghorai S., Bhunia S., Roy M., De D. (2016). Mechanochemical devulcanization of natural rubber vulcanizate by dual function disulfide chemicals. Polym. Degrad. Stab..

[35] Zhang X., Saha P., Cao L., Li H., Kim J. (2018). Devulcanization of waste rubber powder using thiobisphenols as novel reclaiming agent. Waste Manag..

[36] Zedler L., Klein M., Saeb M.R., Colom X., Canavate J., Formela K. (2018). Synergistic Effects of Bitumen Plasticization and Microwave Treatment on Short-Term Devulcanization of Ground Tire Rubber. Polymers.

